# Deciphering cancer cell state plasticity with single-cell genomics and artificial intelligence

**DOI:** 10.1186/s13073-024-01309-4

**Published:** 2024-02-26

**Authors:** Emily Holton, Walter Muskovic, Joseph E Powell

**Affiliations:** 1 Garvan Weizmann Centre for Cellular Genomics, Garvan Institute of Medical Research, The Kinghorn Cancer Centre, Darlinghurst, NSW 2010 Australia; 2 School of Biomedical Science, Faculty of Medicine UNSW Sydney, Kensington, NSW 2010 Australia; 3 UNSW Cellular Genomics Futures Institute, University of New South Wales, Sydney, NSW 2052 Australia

## Abstract

Cancer stem cell plasticity refers to the ability of tumour cells to dynamically switch between states—for example, from cancer stem cells to non-cancer stem cell states. Governed by regulatory processes, cells transition through a continuum, with this transition space often referred to as a cell state landscape. Plasticity in cancer cell states leads to divergent biological behaviours, with certain cell states, or state transitions, responsible for tumour progression and therapeutic response. The advent of single-cell assays means these features can now be measured for individual cancer cells and at scale. However, the high dimensionality of this data, complex relationships between genomic features, and a lack of precise knowledge of the genomic profiles defining cancer cell states have opened the door for artificial intelligence methods for depicting cancer cell state landscapes. The contribution of cell state plasticity to cancer phenotypes such as treatment resistance, metastasis, and dormancy has been masked by analysis of ‘bulk’ genomic data—constituted of the average signal from millions of cells. Single-cell technologies solve this problem by producing a high-dimensional cellular landscape of the tumour ecosystem, quantifying the genomic profiles of individual cells, and creating a more detailed model to investigate cancer plasticity (Genome Res 31:1719, 2021; Semin Cancer Biol 53: 48-58, 2018; Signal Transduct Target Ther 5:1-36, 2020). In conjunction, rapid development in artificial intelligence methods has led to numerous tools that can be employed to study cancer cell plasticity.

## Background

Our understanding of cancer biology will be advanced by resolving the heterogeneity of cancer cells and their relationship with the surrounding microenvironment. While a recent focus has been on the cells comprising the tumour microenvironment, evidence has been building on how cancer cell state plasticity impacts cancer evolution and clinical outcomes [1, 2]. Genetic factors contributing to cancer cell state plasticity are well established, but evidence is emerging for the involvement of transcriptomic and epigenetic mechanisms. As such, cancer cell plasticity should be viewed as a consequence of genetic, transcriptional, and epigenetic mechanisms that promote the continuous switching of cell states [3]. Below, we explore the progress in artificial intelligence methods and how they can help us understand cancer cell state plasticity.

## Static models—creating a landscape of cell states

The data produced by single-cell technologies has several unique features that present analytical challenges: its structure is highly dimensional, with information on thousands of genes across thousands of cells, and it is typically sparse, with a gene-by-cell matrix containing ‘zeros’ for non-expressed genes. These features mean that traditional data visualisation and dimension reduction methods are unsuitable. Manifold learning has become a standard tool in analysing and visualising single-cell data, where a series of techniques use non-linear dimensionality reduction to project the high-dimension cellular landscape onto a low-dimensional “manifold” representation of each cell, retaining complex local relationships and the data’s global structure.

One of the most common manifold learning techniques now applied to single-cell data is Uniform Manifold Approximation and Projection (UMAP). While UMAP is predominately used as a visualisation tool, it can improve secondary analyses, such as cell clustering and annotations, which aid the investigation of possible unknown or uncharacterised cell states. Clustering has been a popular approach in identifying populations of cancer cell states as well as cell states of the tumour microenvironment [4]. However, applying clustering requires careful consideration for downstream analyses, as different cell types can cluster together and, consequently, be annotated as a single cell type. The consequence is that biological information is masked, limiting secondary analyses on the phenotypic impact of cell state variation. A solution is annotating cells on a ‘per-cell’ basis, which solves many challenges but typically requires a reference map to align against.

Manifold learning techniques, conceptionally and practically, lend themselves well to the challenges of understanding cancer cell state plasticity, as they can represent a cell landscape that is both nonlinear and continuous. Cancer plasticity models have used this information to investigate the landscape of cancer cell states by placing cells within an *N*-dimensional landscape. Based on the conceptualisation that cell states can transition, it is possible to analyse the genomic profiles of cells to predict their movement from one position in the landscape to another. This can be extended to consider spaces within this landscape where cells represent ‘stable states’ and the corridors between stable state positions represent transitional routes as cell states become plastic, transiting to another cell state [5].

## Dynamic models—predicting the transitions of cell states

While static models present a snapshot of a cell’s genomic state at the time of capture, artificial intelligence models have been developed to infer the trajectories of cell states—i.e. estimate the future of a cell’s state. One of the first algorithms developed to infer trajectory, *Monocle*, utilises single-cell RNA-seq data to identify gene expression changes along a cell’s presumed transition between states, calculating a pseudotime trajectory for each cell. Since its release in 2014, new methods have been developed to improve the trajectory analysis of biological systems [6].

While trajectory inference allows us to predict the future state of cells, algorithms have been proposed to infer the probability or speed of cell state transition, often called cell velocity. Software such as *Velocyto* and *scVelo*, developed on top of the RNA velocity framework [7], model RNA maturation to predict the rate of cell-state transitions. RNA velocity algorithms are frequently used to investigate cell-state trajectories of cancer cells, advancing our knowledge of tumour systems. One valuable contribution is lineage estimation, which is used to identify divergences in malignant cell lineages between cancer subtypes. Accurately identifying divergent lineages can be a useful clinical tool to predict tumour-specific features such as progression and treatment resistance [7]. Glioblastoma is a clear example of where cell state and dynamic lineage trajectories can be estimated from single-cell data and used to infer cancer cell developmental processes arising from specific cell states [8].

## Deep learning—opportunities to infer biological function

Implementing deep learning technologies, a subgroup of artificial intelligence, has improved the identification of cell states and estimation of state transitions [9]. An early challenge in applying deep learning algorithms was their ability to scale to the size and dimensionality of single-cell data. With the improvement of software and hardware and parallelisation of algorithms, this challenge has now largely been overcome, and the use of deep learning methods in profiling cancer cell state plasticity is a rapidly developing area.

It has been demonstrated that deep learning methods can be more accurate than static models for estimating the overall landscape of cells and, by extension, positioning each cell within that landscape. Autoencoder methods, originally used to perform quality control and clustering, have been extended to ‘deconvolute’ biological signals from noise in single-cell data. Applied to problems of cancer cell state plasticity, autoencoder methods can construct manifold representations of the data and place cells within that space using a subset of the data representing specific biological features [9]. This is typically represented in the ‘bottleneck layer’, which captures the encoding pertinent to the biological feature, allowing these trained features to be projected onto independent datasets [5, 9]. A related technique, archetypal analysis, is an alternative approach for identifying groups of cells that share the same ‘archetype’, which can be thought of as a cell state profile. In cancer, these methods can be trained to identify different cell archetypes representing a particular cell state or transition. As a result, we can avoid labelling cells and instead place them within a continuous landscape, enabling the investigation of cell state dynamics and identifying intermediate cell states. This is valuable, as we expect that, within a tumour, cancer cells may be transitioning between states, and by identifying them, we can learn about the genomic programmes underlying cancer cell state plasticity.

With deep learning methods an evolving and promising field, one of the challenges is interpreting meaningful biological information from the analysis. Work addressing this issue is emerging, with knowledge-primed neural networks used to demonstrate the flow of genomic information in cell states and improve interpretability [10]. In knowledge-primed neural networks, each node within the model corresponds to a gene or protein, and each edge corresponds to a regulatory relationship previously observed in annotated data. Weightings can be applied based on biological priors, such as estimating the regulatory importance—for example, how important is a transcription factor or signalling protein to the biological problem? This approach has been used to investigate tumour systems where cancer cell state plasticity is known to underly tumour heterogeneity [10].

## Conclusions

Single-cell genomics provides high-resolution and high-dimensional characterisation of heterogeneous cells in a tumour. Combined with artificial intelligence, we now have the potential to identify cancer cell states as well as infer their plasticity between states. Because of the depth of genomic data available, accurately identifying these states and state transitions and separating the relevant biological signals from noise provides a powerful approach to determining the cause of cancer cell plasticity and the consequence of state changes. Given the impact of cell plasticity on cancer cell phenotypes and behaviours, this knowledge will inform the development of treatment strategies tailored to the unique characteristics of a patient’s cancer cell states, improving outcomes for cancer patients and contributing to an improved understanding of cancer heterogeneity.

## Data Availability

Not applicable.
